# MAP7D3, a novel prognostic marker for triple-negative breast cancer, drives cell invasiveness and cancer-initiating cell properties to promote metastatic progression

**DOI:** 10.1186/s13062-023-00400-x

**Published:** 2023-08-07

**Authors:** Wen-Hung Kuo, Pei-Yi Chu, Chen-Chi Wang, Ping-Shen Huang, Shih-Hsuan Chan

**Affiliations:** 1 Department of Surgery, National Taiwan University Hospital, Taipei, 100 Taiwan; 2 Department of Post-Baccalaureate Medicine, College of Medicine, National Chung Hsing University, Taichung, 402 Taiwan; 3 School of Medicine, College of Medicine, Fu Jen Catholic University, New Taipei City, 242 Taiwan; 4 Department of Pathology, Show Chwan Memorial Hospital, Changhua, 500 Taiwan; 5 National Institute of Cancer Research, National Health Research Institutes, Tainan, 704 Taiwan; 6 Department of Nutrition, China Medical University, Taichung, 40402 Taiwan; 7 School of Chinese Medicine, College of Chinese Medicine, China Medical University, Taichung, 40402 Taiwan; 8 Cancer Biology and Precision Therapeutics Center, China Medical University, Taichung, 40402 Taiwan; 9 Chinese Medicine Research Center, China Medical University, Taichung, 40402 Taiwan

**Keywords:** Triple-negative breast cancer, Metastasis, MAP7D3, Breast cancer-initiating cells.

## Abstract

**Background:**

Patients with triple-negative breast cancer (TNBC) tend to develop visceral metastasis within five years, making them the most challenging BC patients to treat. The MAP7 protein family is a group of microtubule-binding proteins with a well-known role in microtubule-related cell migration, but its role in metastasis-related properties of TNBC remains unclear.

**Methods:**

qRT-PCR and western blot were used to validate mRNA and protein expression of the MAP7 family in the isogenic pairs of TNBC cell lines with low and high metastasis potential. Functional characterization of MAP7D3 was carried out using cell-based and mouse models. The clinical association between MAP7D3 and TNBC was established using datasets in the public domain.

**Results:**

MAP7D3 expression was consistently upregulated in the metastatic subline IV2 and 468-LN at both mRNA and protein levels. Knockdown of MAP7D3 inhibited the 3D colony-forming ability, cell migration, and invasion ability of IV2 and 468-LN, indicating its significant contribution to the metastasis phenotypes. Mechanistically, inhibition of MAP7D3 could significantly increase the sensitivity of metastatic TNBC cells to docetaxel and gemcitabine treatment by reducing the expression of proteins related to breast cancer-initiating cells (BCICs) and drug resistance, as well as suppressing the activity of Rac1. The animal study showed that the depletion of MAP7D3 drastically reduced TNBC tumor growth and impaired the metastatic capability of TNBC cells. Elevated expression of MAP7D3 was found in the metastatic lymph nodes and was significantly associated with advanced stage and higher grade TNBC. Moreover, MAP7D3 expression was significantly correlated with the TNBC population, and its high expression was significantly associated with lymph node metastasis and poor survival outcomes of patients with TNBC.

**Conclusion:**

Our study indicates that targeting MAP7D3 could be a promising therapeutic strategy for addressing the progression of TNBC, and MAP7D3 may serve as a novel predictive biomarker for the survival outcomes of triple-negative breast cancer.

**Supplementary Information:**

The online version contains supplementary material available at 10.1186/s13062-023-00400-x.

## Introduction

Triple-negative breast cancer (TNBC) is considered the most difficult-to-treat BC subtype because patients with TNBC do not respond to the current standard treatments including hormone therapy and HER2 targeted therapy and generally have poorer response to the standard chemo drugs. Therefore, patients with TNBC have higher tendency to have metastatic disease within five years after diagnosis as compared to other BC subtypes. Recently, the PARP inhibitor, a cancer drug targeting the poly (ADP-ribose) polymerase, has been approved by U.S FDA to use in patients with BRCA1/2 mutations. However, BRCA1/2 mutations occur only in less than 10% of TNBC population [1–3] and are not related to the metastatic spread of TNBC.

During the tumor progression, the hypoxia and the lack of nutrients are two well-known factors that drive tumor cells inside the tumor mass to undergo the “epithelial-to-mesenchymal transition (EMT)”, a drastic cellular transformation that allows tumor cells to acquire motility to escape the primary site. To undergo such drastic physical transformation, the cytoskeleton molecules responsible for cell migration such as actin filaments and microtubules need to be re-organized to form the lamellipodia and the filipodia which could enable tumor cells to gain invasive properties. TNBC cells with EMT phenotype have been shown to be highly associated with chemoresistance [4]. According to previous studies, breast cancer-initiating cells (BCICs) expressing specific marker genes including CD44, ALDH1A1, ABCG2, Integrin α6, Sox2, and EpCAM have been identified as the main population responsible for chemoresistance in TNBC cells exhibiting EMT phenotype [5, 6].

The MAP7 (Microtubule Associated Protein 7) protein family, consisting of four members, MAP7, MAP7D1 (MAP7 Domain Containing 1), and MAP7D2 (MAP7 Domain Containing 2), MAP7D3 (MAP7 Domain Containing 3), is the microtubule-associated protein involved in various cellular processes regulating microtubule dynamics, organization, and stability [7, 8]. By stabilizing microtubules, MAP7 could enhance neuronal branch and control the length of microtubules in the mitotic spindle [9–11]. Kikuchi et al., showed that MAP7 and MAP7D1 bind to microtubules were required for cell-substrate adhesion and migration in HeLa cells and inhibition of MAP7 and MAP7D1 impaired cancer cell adhesion and migration [12]. However, whether MAP7 family is involved in TNBC progression is currently unknown.

In this study, we sought to address this gap in knowledge by investigating the role of MAP7 family in TNBC progression as well as its potential association with clinical outcomes of patients with TNBC. By analyzing the expression levels of MAP7 family in two isogenic pairs of TNBC cells with low and high metastatic potential, MDA-MB-231-P/231-IV2 and MDA-MB-468-P and 468-LN, we showed that MAP7D3 was consistently overexpressed in the metastatic sublines as compared to the respective parental lines. Functional assays indicated that depletion of MAP7D3 expression significantly inhibited the ability of cancer cell migration, invasion, and 3D colony formation. Mechanistically, we demonstrated that depletion of MAP7D3 significantly impaired Rac1 activity and inhibited BCICs marker expressions. Furthermore, inhibition of MAP7D3 significantly sensitized the metastatic sublines to docetaxel and Gemcitabine treatment. Our animal study showed that MAP7D3 depletion effectively suppressed TNBC tumor growth and metastatic progression in the orthotopic TNBC mouse model. Clinically, we demonstrated that high levels of MAP7D3 expression were significantly correlated with TNBC among other breast cancer subtypes. Furthermore, we found that increased expression of MAP7D3 was significantly associated with late-stage TNBC, lymph node metastasis, and poor survival outcomes in TNBC patients.

## Results

### MAP7D3 expression is significantly upregulated in the highly metastatic TNBC cells

**Fig. 1:**
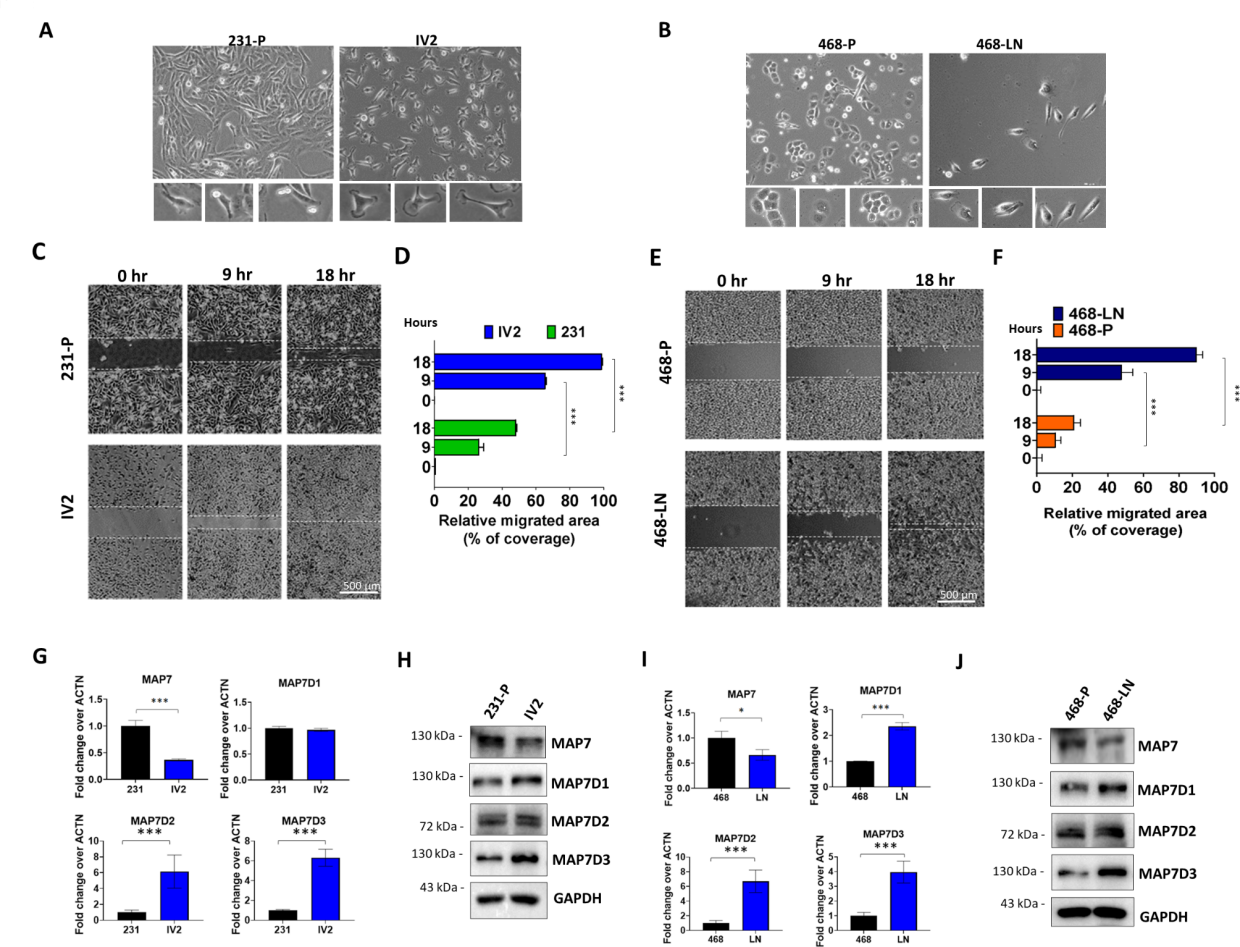
Identification of isogenic pair of TNBC cell lines with low and high motility. (**A**) Cell morphology of MDA-MB-231-P and IV2 cells under light microscopy (100X). The lower panel is the enlargement of single cell image from the upper panel image. (**B**) Cell morphology of MDA-MB-468-P and 468-LN cells under light microscopy (100X). The lower panel is the enlargement of single cell image from the upper panel image. (**C**) Time lapse of wound healing assay. Scale bar, 500 μm. (**D**) Quantitative results of migrating area between 231-P and IV2. (**E**) Time lapse of wound healing assay. Scale bar, 500 μm. (**F**) Quantitative results of migrating area between 468-P and 468-LN. *** P < 0.001. (**G**) qRT-PCR analysis of MAP7, MAP7D1, MAP7D2, MAP7D3 in 231-P and IV2. (**H**) Western blotting analysis of MAP7D1, MAP7D2 and MAP7D3 in 231-P and IV2. (**I**) qRT-PCR analysis of MAP7, MAP7D1, MAP7D2, MAP7D3 in 468-P and 468-LN. (**J**) Western blotting analysis of MAP7D1, MAP7D2 and MAP7D3 in 468-P and 468-LN. *** P < 0.001. *P < 0.05. Each experiment was performed in triplicate and repeated three times

First, we analyzed the expression levels of MAP7 family in two isogenic pairs of TNBC cells with low and high metastasis potential. The highly metastatic IV2 cells were derived from the TNBC cell line MDA-MB-231 using in vivo selection protocol [13]. In this study, we further generated another highly metastatic subline 468-LN derived from the lymph node metastasis in a MDA-MB-468 xenograft mouse model. As shown in Fig. 1A and B, morphologically, IV2 and 468-LN displayed spindle-like cell shape and large lamellipodia structures as compared to their respective parental lines, MDA-MB-231 and MDA-MB-468. First, the motility of the highly metastatic subline IV2 and 468-LN was validated using wound-healing assay. The results showed that IV2 and 468-LN cells both displayed increased cell migration as compared to their respective parental line (Fig. 1C-F). Next, the expression of MAP7, MAP7D1, MAP7D2 and MAP7D3 was investigated in two isogenic pairs of TNBC cells. We showed that, among MAP7 members, only MAP7D3 was consistently upregulated at both mRNA and protein levels in IV2 and 468-LN cells as compared to their respective parental cells (Fig. 1G-J). Thus, higher expression of MAP7D3 was confirmed in the highly metastatic TNBC cells.

### MAP7D3 depletion leads to the suppression of in vitro metastasis traits of the highly metastatic TNBC cells

**Fig. 2:**
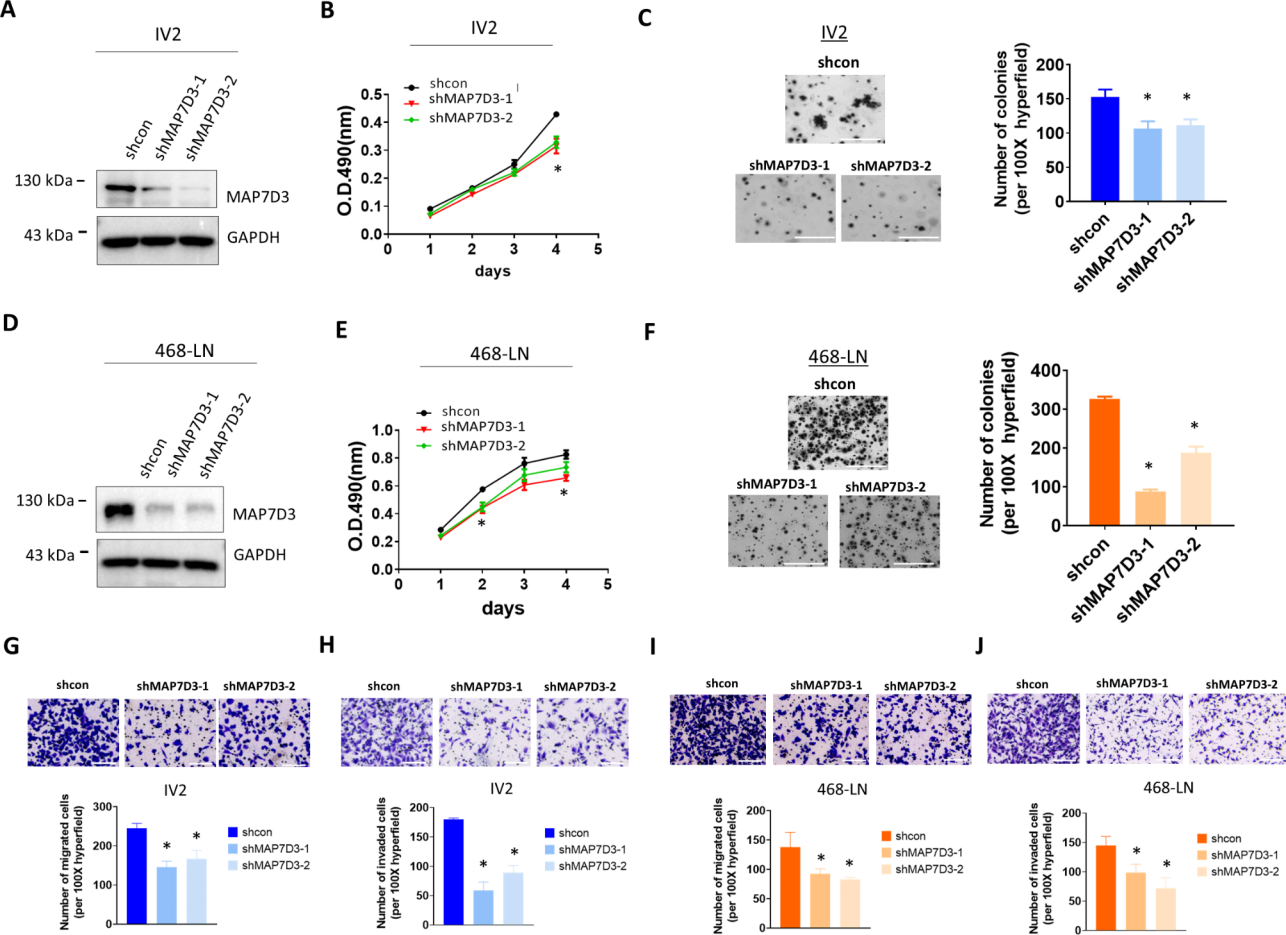
Depletion of MAP7D3 impairs in vitro metastasis traits of the highly metastatic IV2 and 468-LN cells. **(A)** Western blotting analysis of MAP7D3 protein expression in the control IV2 cells and the stable MAP7D3 knockdown IV2 cells. **(B)** Cell proliferation of the control IV2 cells and the stable MAP7D3 knockdown IV2 cells. **(C)** Soft agar assay of the control IV2 cells and the stable MAP7D3 knockdown IV2 cells. Scale bar, 1000 μm. **(D)** Western blotting analysis of MAP7D3 protein expression in the control 468-LN cells and the stable MAP7D3 knockdown 468-LN cells **(E)** Cell proliferation analysis of the control 468-LN cells and the stable MAP7D3 knockdown 468-LN cells. **(F)** Soft agar assay of the control 468-LN cells and the stable MAP7D3 knockdown 468-LN cells. Scale bar, 1000 μm. **(G)** Tranwell migration assay of the control IV2 cells and the stable MAP7D3 knockdown IV2 cells. Scale bar, 200 μm. **(H)** Matrigel-coated Tranwell assay of the control IV2 cells and the stable MAP7D3 knockdown IV2 cells. Scale bar, 200 μm. **(I)** Tranwell migration assay of the control 468-LN cells and the stable MAP7D3 knockdown 468-LN cells. Scale bar, 200 μm. **(J)** Matrigel-coated Tranwell migration assay of the control 468-LN cells and the stable MAP7D3 knockdown 468-LN cells. Scale bar, 200 μm. * P < 0.05. Each experiment was performed in triplicate and repeated three times

Next, in order to understand whether MAP7D3 expression could contribute to the aggressive phenotype of IV2 and 468-LN cells, we established stable MAP7D3 knockdown cells from IV2 and 468-LN cells using shRNA-expressing lentivirus approach. As shown in Fig. 2A and D, MAP7D3 expression was successfully depleted by two MAP7D3 shRNAs in IV2 and 468-LN cells. We found depletion of MAP7D3 in IV2 cells impaired cell growth in the MTS cell proliferation assay (Fig. 2B). Similar results were observed in 468-LN cells that depletion of MAP7D3 also led to the decrease of cell proliferation (Fig. 2E). Notably, we found that MAP7D3 depletion could significantly reduce the anchorage-independent growth in the soft agar assay in both IV2 and 468-LN cells (Fig. 2C-F). Next, we tested if MAP7D3 depletion could affect cell motility and invasiveness of the highly metastatic cells. Transwell migration assay showed that depletion of MAP7D3 greatly impaired the ability of cell migration of IV2 and 468-LN cells (Fig. 2G and I). Similarly, we found both MAP7D3-depleted IV2 cells and MAP7D3-depleted 468-LN cells displayed reduced invasiveness as compared to their respective control cells in the Matrigel-coated invasion assay (Fig. 2H-J). Collectively, our results showed that MAP7D3 significantly contributes to the aggressive phenotype of TNBC cells.

### Inhibition of MAP7D3 reduces breast cancer-initiating cell markers and Rac1 activity in metastatic TNBC cells and significantly sensitizes cells to docetaxel and gemcitabine treatment

**Fig. 3:**
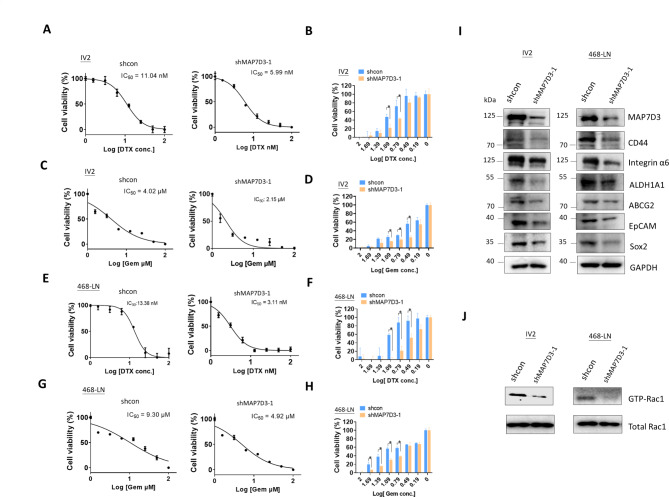
MAP7D3 depletion significantly sensitizes TNBC cells to docetaxel/gemcitabine treatment and reduces breast cancer initiating cells (BCICs)-associated protein marker expression. **(A)** Determination of the IC50 of docetaxel in the control IV2 cells and the shMAP7D3 IV2 cells. **(B)** Statistical analysis of the IC50 of docetaxel between the control IV2 cells and the shMAP7D3 IV2 cells. *P* < 0.05. **(C)** Determination of the IC50 of gemcitabine in the control IV2 cells and the shMAP7D3 IV2 cells **(D)** Statistical analysis of the IC50 of docetaxel between the control IV2 cells and the shMAP7D3 IV2 cells. *P* < 0.05. **(E)** Determination of the IC50 of docetaxel in the control 468-LN cells and the shMAP7D3 468-LN cells. **(F)** Statistical analysis of the IC50 of docetaxel between the control 468-LN cells and the shMAP7D3 468-LN cells. *P* < 0.05. **(G)** Determination of the IC50 of gemcitabine in the control 468-LN cells and the shMAP7D3 468-LN cells. **(H)** Statistical analysis of the IC50 of gemcitabine between the control 468-LN cells and the shMAP7D3 468-LN cells. *P* < 0.05. **(I)** Western blotting analysis of breast cancer initiating cells (BCICs) protein marker expressions in the control cells and shMAP7D3 cells. **(J)** Western blotting analysis of GTP-Rac1 pull-down in the control cells and shMAP7D3 cells. Each in vitro experiment was performed in triplicate and repeated three times

As chemotherapy resistance frequently developed in patients with TNBC, we investigated whether depleting MAP7D3 could affect the treatment response of standard chemo drugs docetaxel and gemcitabine between the control TNBC cells and MAP7D3-depleted TNBC cells. As shown in Fig. 3A and B, the IC50 values for the cytotoxic effect of docetaxel in the control IV2 cell and MAP7D3-depleted IV2 cells were 11.01 nM and 5.99 nM, respectively. On the other hand, the IC50 values of gemcitabine in the control IV2 cells and MAP7D3-depleted IV2 cells were 4.02 µM and 2.15 µM, respectively (Fig. 3C and D). The IC50 of docetaxel and gemcitabine in 468-LN was also determined. As shown in Fig. 3E and F, the IC50 values of docetaxel in the control 468-LN cells and MAP7D3-depleted 468-LN cells were 13.38 nM and 3.11 nM, respectively. In addition, the IC50 values of gemcitabine in the control 468-LN cells and MAP7D3-depleted 468-LN cells were 9.30 µM and 4.92 µM, respectively (Fig. 3G and H). Our IC50 experiments indicated that depletion of MAP7D3 could increase chemosensitivity of TNBC cells to docetaxel and gemcitabine treatment by 2–3 fold and approximately 2-fold, respectively. Next, we investigated if MAP7D3 depletion could alter the expression of proteins related to chemoresistance phenotype of breast cancer. Here, we examined an array of proteins known for contributing the phenotype of breast cancer chemoresistance and relevant for cancer-initiating cells (CICs). The results of western blotting showed that depletion of MAP7D3 in both IV2 and 468-LN cells significantly reduced the expression of integrin-α6, ALDH1A1 (Aldehyde Dehydrogenase 1 Family Member A1), ABCG2 (ATP-binding cassette (ABC), sub-family G, isoform 2 protein), EpCAM (Epithelial Cell Adhesion and Activating Molecule), and Sox2 (Sex Determining Region Y-box 2) as compared to the respective control cells (Fig. 3I). These results further explained the increased chemosensitivity observed in MAP7D3-depleted TNBC cells in response to docetaxel and gemcitabine treatment (Fig. 3A-H). Additionally, microtubule dynamics has been found to be essential for the regulation of actin filament polymerization, which governs by small GTPase activity [14]. Therefore, we examined the inhibition of MAP7D3 could affect Rac1 activity. As expected, GTP-Rac1 pull-down assay showed that depletion of MAP7D3 led to the reduction of Rac1 activity in metastatic TNBC cells (Fig. 3J).

These results indicated that the depletion of MAP7D3 could effectively reduce the expression of proteins related to cancer-initiating cells (CICs) and chemotherapy resistance, as well as decrease Rac1 activity in TNBC cells. Consequently, these findings suggested that MAP7D3 depletion could promote drug sensitivity in TNBC cells.

### Depletion of MAP7D3 expression inhibits TNBC primary tumor growth and lung metastatic capability in a TNBC xenograft mouse model

**Fig. 4:**
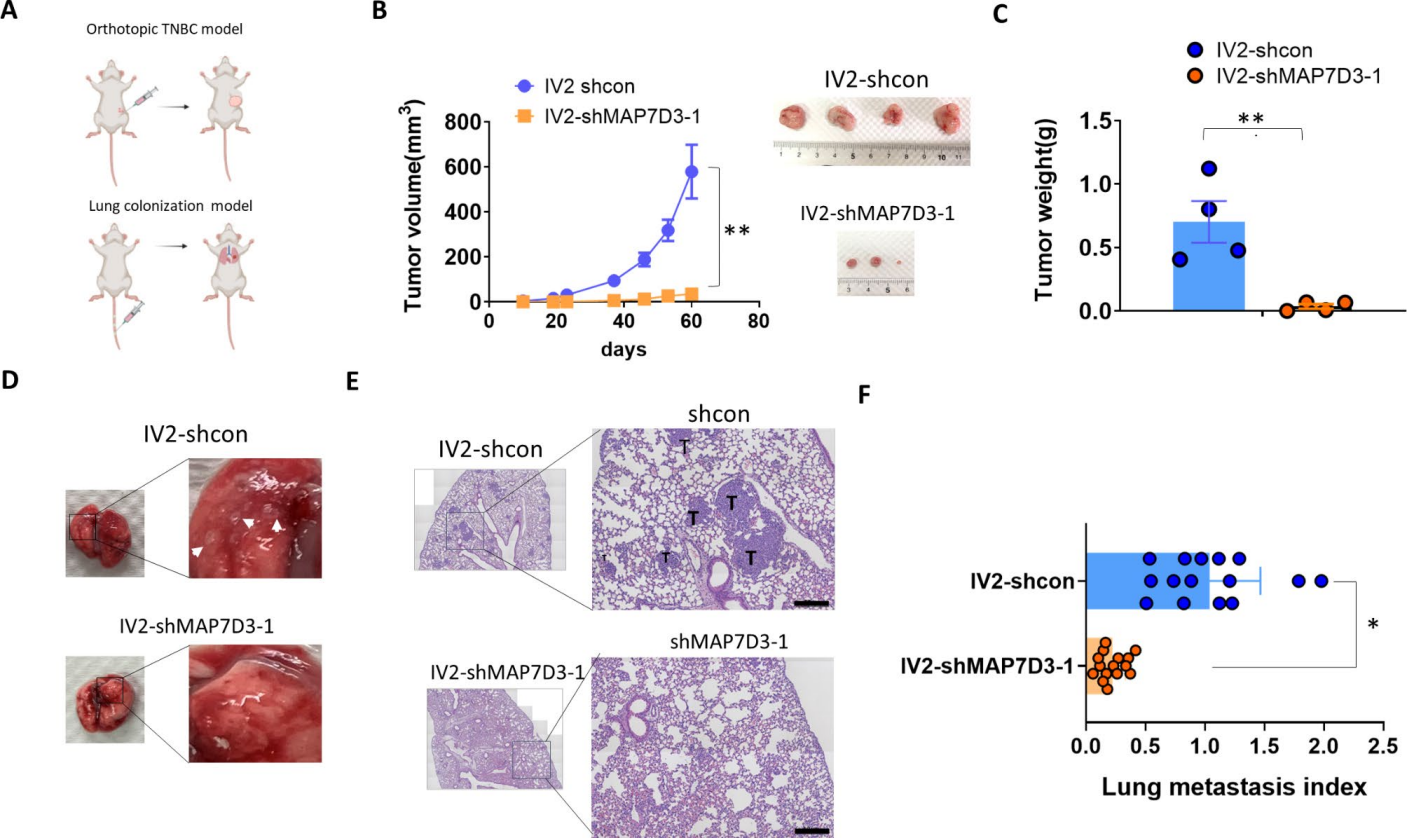
Inhibition of MAP7D3 expression suppresses TNBC primary tumor growth and lung metastatic capability in a mouse model. **(A)** Schematics of the orthotopic and lung colonization mouse model. **(B)** Tumor growth kinetics of tumor-bearing mice. **P < 0.001. **(C)** Tumor weight analysis of tumors resected from the orthotopic mouse model. **P < 0.001. **(D)** The representative images of mouse lungs resected from the lung colonization model. White arrows indicated the metastatic lung nodules. **(E)** The representative HE staining images of mouse lung section from mice injected with the control cells and shMAP7D3 cells, respectively. M35etastatic tumors were indicated by the uppercase T. Scale bar, 50 μm. **(F)** The quantitative results of lung metastasis. Lung metastasis index was calculated as follows: lung parenchyma area that was taken up by metastatic tumors divided by the total area of lung parenchyma. **P < 0.001, *P < 0.05

As aforementioned, inhibition of MAP7D3 expression significantly suppressed the metastasis-related capacities of TNBC cells in vitro, we next investigated if MAP7D3 depletion affected TNBC progression in a mouse model (Fig. 4A). First, the control IV2 cells and MAP7D3-depleted IV2 cells were orthotopically injected into the mouse fat pad and the tumor growth kinetics was analyzed for 8 weeks. As shown in Fig. 4B, tumor growth kinetics analysis showed that MAP7D3 depletion effectively inhibited tumor growth of IV2 cells. Mice injected with the MAP7D3-depleted IV2 cells grew smaller tumors than those mice injected with the control IV2 cell (Fig. 4C and B). Additionally, we examined if inhibition of MAP7D3 could alter the capacity of distant lung metastasis of IV2 cells. To do so, the control IV2 cells and MAP7D3-depleted IV2 cells were injected into the SCID mice via tail vein, respectively, and mice were sacrificed four weeks post cancer cell injection for the examination of lung metastasis status. The result of tail vein injection experiment demonstrated that MAP7D3-depleted IV2 cells failed to form metastatic nodules at mouse lung as compared to the control IV2 cells, which displayed a potent lung metastasis capability (Fig. 4D-F). Together, our mouse experiment demonstrated that MAP7D3 plays a significant role in the progression of TNBC. Specifically, inhibiting the expression of MAP7D3 resulted in a substantial impairment of primary tumor growth and a decrease in the potential for distant lung metastasis in metastatic TNBC cells.

### High expression of MAP7D3 correlates with lymph node metastasis and poorer survival outcomes of triple-negative breast cancer

**Fig. 5:**
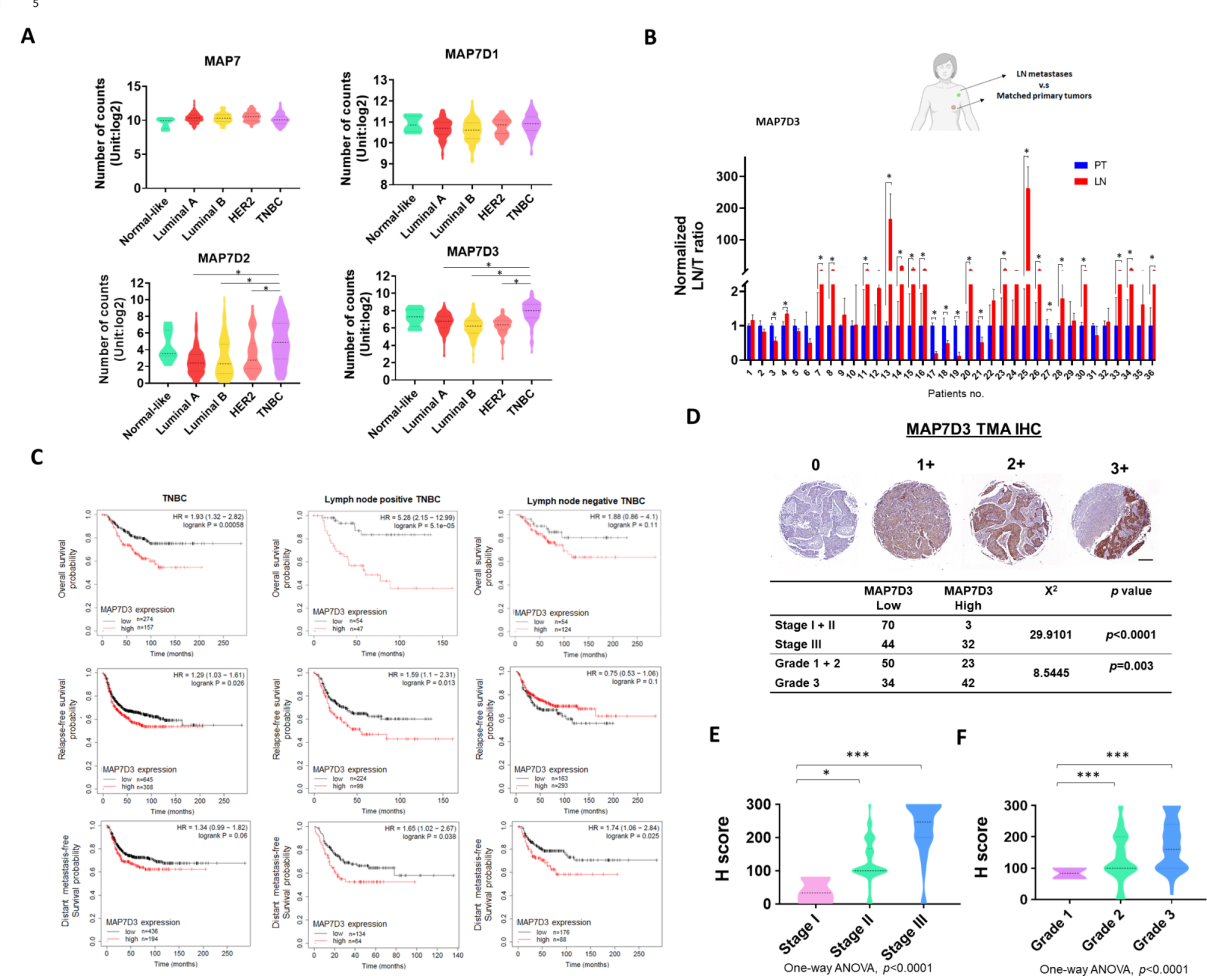
High expression of MAP7D3 predicts poor survival outcomes of triple-negative breast cancer (TNBC) patients and is highly associated with lymph node metastatic tumors and late-stage TNBC. (**A**) Analysis of the expression of MAP7 family among BC subtypes in TCGA breast cancer dataset using UCSC Xena platform. *P < 0.05. Normal-like, n = 8; Luminal A, n = 231; Luminal B, n = 127; HER2. n = 58; TNBC, n = 98). (**B**) Collection of the lymph node (LN) metastatic tumors and the primary tumors from the same patient. Quantitative reverse transcription-PCR (qRT-PCR) analysis of MAP7D3 mRNA expression in the LN metastatic tumors and the corresponding primary tumors. *P < 0.05. (**C**) Analysis of survival outcomes of TNBC patients with or without lymph node metastasis according to MAP7D3 expression was performed using the Kaplan-Meier method in GSE TNBC datasets. Overall survival, relapse-free survival, and distant metastasis-free survival of TNBC patients were estimated based on the best cutoff value of MAP7D3 expression. The survival status was stratified by lymph node positivity of TNBC. (**D**) Analysis of the correlation between MAP7D3 expression and TNBC stages/grades using Pearson’s Chi-square test. (**E**) H score of SCEL IHC staining versus TNBC stages. stage I, n = 3; stage II, n = 110; stage III, n = 36. *P < 0.05, ***P < 0.001. (**F**) H score of SCEL IHC staining versus TNBC grades. grade 1, n = 2; grade 2, n = 82; grade 3, n = 65. ***P < 0.001

Next, we investigated the clinical relevance of MAP7 family in BC clinical databases from public domain. First, we analyzed the mRNA expression levels of all MAP7 members in TCGA breast cancer dataset (TCGA-BRCA) using the UCSC Xena platform [15]. The results showed that MAP7D2 and MAP7D3 were significantly correlated with TNBC population, which is the most aggressive subtype among BC subtypes (Fig. 5A). Next, we examined the mRNA levels of MAP7D3 in our cohort samples containing 36 paired lymph node (LN) metastasis tissues and primary tumor tissues (Fig. 5B). qRT-PCR results showed that the expression of MAP7D3 was upregulated in 63.8% of LN metastatic tumors (23/36) when compared with the matched primary tumors (Fig. 5B), indicating that MAP7D3 plays an essential role in TNBC metastatic progression. Furthermore, we explored the correlation between MAP7D3 expressions and the survival outcomes of TNBC patient in 48 GSE clinical TNBC datasets using KM plotter [16] (Fig. 5C). Our analyses showed that high expression of MAP7D3 was significantly associated with poorer survival rate of TNBC patients in three clinical survival categories: overall survival (OS), relapse-free survival (RFS), and distant metastasis-free survival (DMFS) (Fig. 5C). Notably, when stratified by LN positivity, LN-positive patients with high expression of MAP7D3 had the worst OS and RFS (Fig. 5C). Additionally, we performed IHC staining in TNBC tissue microarrays to establish the link between MAP7D3 protein expression and disease progression (Fig. 5D). The Chi-square analysis indicated that there was a significant association between high expression of MAP7D3 and late-stage (Fig. 5D, p < 0.0001) as well as higher grade of TNBC (Fig. 5D, p = 0.003). The result of IHC staining showed that high expression was significantly associated with the late stage and higher grade of TNBC (Fig. 5E F), indicating that MAP7D3 expression significantly correlates with TNBC disease progression.

Collectively, our clinical findings provide first evidence to demonstrate the clinical relevance of MAP7D3 in TNBC that high expression of MAP7D3 is associated with tumor progression, LN metastasis, and poor survival outcomes of TNBC patients.

## Materials and methods

### Cell culture

MDA-MB-231-P, 231-IV2, MDA-MB-468, and MDA-MB-468-LN were maintained in the 10% FBS (fetal bovine serum) DMEM (Dulbecco’s Modified Eagle Medium) supplemented with 1% penicillin/streptomycin and 2 mM L-glutamine and cultured under the air condition of 5% CO_2_ in the 37℃ humidified incubator. Cell lines were routinely tested for mycoplasma contamination. All cell lines used in this study were authenticated by STR profiling.

### In vivo selection of highly metastatic TNBC cells

2.5 million MDA-MB-468 cells were orthotopically injected into the 2nd fat pad of SCID mouse, and the tumor growth and the subsequent lymph node metastasis was monitored for four months. Lymph node tumors were then resected and digested with trypsin and accutase for one hour in the 37℃ humidified incubator in order to isolate the metastatic cells. The resulting 468-LN subline was then continued to culture for one month followed by STR authentication. The establishment of highly metastatic 231-IV2 cells was described in our previous work [13].

### Transwell migration and invasion assay

The 24-well 8 μm Tranwells were placed on the 24-well plate prior to the experiment. Next, 1 × 10^4^ cells were resuspended in 300 µl 0.1% BSA DMEM, and seeded onto the Transwell followed by adding 0.5 mL 10% DMEM to the bottom chamber. Cells were further cultured for 8 h. After 8 h of incubation, cells that migrated through 8 μm porous membrane were fixed and stained with 0.1% crystal violet fixation buffer. The images of the migrated cells were taken under microscope. To assess the cancer cell invasiveness, cells were seeded onto the 24-well Matrigel-coated Transwell followed by adding 0.5 mL 10% DMEM to the bottom chamber. Cells were further cultured for 24 h followed by staining with 0.1% crystal violet fixation buffer. The images of the invaded cells were obtained using the light microscope. The images of cells were quantified using Image J software (Image J, CA, USA).

### Soft agar formation assay

1.2% agar was heated in a microwave and kept in a 42℃ water bath. Next, a base agar was created by combining equal volumes of pre-warmed 2X medium with 20% FBS with pre-warmed 1.2% agar. Then, 1.5 mL of this mixture was added to each well of a 6-well plate. To prepare the top agar, 0.6% agar was melted and kept in a 42℃ water bath. Subsequently, 10,000 cells were mixed with 0.75 mL of 2X medium containing 20% FBS and an equal volume of pre-warmed 0.6% agar to yield a final concentration of 0.3% top agar. Once the top agar had solidified, complete medium was added, and the cells were maintained at 37℃ in a humidified incubator for 14 days. Finally, viable cells were stained and visualized using iodonitrotetrazolium chloride (Sigma Aldrich, CA, USA).

### RNA extraction and reverse transcription-quantitative polymerase chain reaction (RT-qPCR)

Cell samples were collected and lysed using 1 mL of TRIzol (Invitrogen, CA, USA). To facilitate phase separation, 200 µL of chloroform was added to the lysate, followed by vigorous shaking to ensure thorough mixing. The mixture was then placed on ice for 5 min. Subsequently, centrifugation was performed to separate the aqueous phase (containing RNA) from the organic phase. Carefully, the aqueous phase was transferred to a new tube. To precipitate the RNA, an equal volume of isopropanol was added to the aqueous phase. The mixture was gently mixed and incubated on ice for 30 min. Further centrifugation was carried out to pellet the RNA, while the supernatant was discarded. To remove any impurities, the RNA pellet was washed with 75% ethanol. Finally, rehydration of the RNA pellet was performed using RNase-free water. 1 µg of RNA was reversed transcribed using Superscript reverse transcriptase following the manufacturer’s instruction (Invitrogene, CA, USA). Quantitative polymerase chain reaction was carried out using the gene-specific primers under the defined thermocycler protocol with KAPA SYBR® FAST qPCR Master Mix (Roche, IN, USA). The gene expressions were determined using ΔΔCT method (2^(-ΔΔCT) where ΔΔCT represents the difference in threshold cycle (CT) values between the experimental sample and the control/reference sample.

### Western blot

Cellular lysis was performed using a lysis buffer consisting of 25mM Tris (pH 7.6), 150mM NaCl, 1% sodium deoxycholate, 1% Triton-X100, and 0.1% SDS. Quantification of protein samples was carried out utilizing a BCA protein assay kit (Thermo Fisher, CA, USA). Subsequently, the protein samples were subjected to SDS-PAGE for separation, followed by transfer onto a PVDF membrane. To prevent nonspecific binding, the PVDF membranes were blocked with 5% non-fat milk at room temperature for one hour. For antibody detection, the PVDF membranes were incubated overnight at 4℃ with specific primary antibodies, followed by hybridization with the corresponding secondary antibodies. Chemiluminescence signals were generated by applying an ECL substrate onto the membrane, and these signals were then detected and quantified using the ChemiDoc imaging system (Biorad, CA, USA).

### Immunohistochemistry staining (IHC)

IHC staining was carried out as the published procedure (25) using anti-MAP7D3 (Fortis Life Sciences, Inc., CA, USA). The results of IHC score of MAP7D3 were determined as follows: 0 score: No observed staining; 1 + score: faint staining in > 10% tumor cells; 2 + score: moderate, homogenous staining > 25% tumor cells. staining; 3 + score: Dark, homogenous staining > 50% tumor cells. The H-score of MAP7D3 was calculated as the staining intensity of cancer cells (0 = none; 1 = weak; 2 = moderate; and 3 = strong) multiplied by the percentage of tumor sections being stained (0–100%). All IHC samples were independently scored by two investigators in a double-blinded manner and were reviewed by a pathologist.

### GTP- Rac1 pull-down assay

Analysis of active Rac1 activity in the control cells and MAP7D3-depleted cells was carried out using Active Rac1 Detection kit (Cell Signaling Technology, CA, USA) according to the manufacturer’s instruction. Briefly, 100 µL of glutathione resin was transferred to a spin cup, and 20 µg of GST-PAK1-PBD was added. Next, 1 mg of total cell lysate was added to the spin cup and incubated at 4 °C for 1 h with gentle rocking. The spin cup was then subjected to centrifugation at 6,000 xg for 30 s. Following centrifugation, the spin cup was washed three times with 1X wash buffer at 6,000 xg for 30 s each. To elute the samples, the reducing sample buffer was added to the spin cup. Finally, the resulting samples were subjected to western blot analysis.

### Determination of IC50 values for docetaxel and gemcitabine in TNBC cells

1.5 × 10^4^ cells were seeded in the 96-well plate 24 h prior to the drug treatment. The control cells and the MAP7D3-depleted cells were treated with docetaxel at dosages ranging from 0 to 100 nM for 48 h followed by MTS assay. The cells were similarly treated with gemcitabine ranging from 0 to 100 nM. The IC50 values for docetaxel or gemcitabine were calculated using GraphPad prism 8 ® (GraphPad software, CA, USA).

### Patients’ information

Thirty-six paired TNBC primary tumors and lymph node metastatic tumors were collected from National Taiwan University Hospital (NTUH). Collection of TNBC patient-derived primary tumors and lymph node metastatic tumors was approved by the Research Ethics Committee at NTUH (Approval number:202104008RINC). TNBC tissue microarrays were purchased from Biomax Inc. (CA, USA).

### Orthotopic TNBC mouse model

One million control IV2 cells and MAP7D3-depleted IV2 cells were injected into the 4th fat pad of SCID mice, respectively. Tumor growth kinetics was recorded until the endpoint of the experiment. Tumor-bearing mice were sacrificed on week eight and tumors were resected for the analysis of tumor weight.

### Lung colonization mouse model

0.5 million control IV2 cells and MAP7D3-depleted IV2 cells were injected into the SCID mice via tail vein, respectively. Mice were then sacrificed four weeks post cancer cell injection. The mouse lungs were resected, and fixed in 4% paraformaldehyde for HE staining analysis. The lung metastasis nodules were examined and photographed under the light microscope. The lung metastatic area was determined using Image J software (CA, USA).

### Public domain datasets

The expression of MAP7 family among BC subtypes was investigated in the TCGA breast cancer (BCRA) dataset using UCSC Xena online platform (www.ucscxena.com). A total of 48 GSE datasets (see supplementary file) was used to analyze the correlation of MAP7D3 expression with survival outcome in TNBC population using KM plotter online platform (www.kmplot.com). TNBC samples were extracted from GSE datasets based on PAM50 for survival analysis. The cutoff value of MAP7D3 was determined using the function of auto select best cutoff [17], where all possible cutoff values between the lower and upper quantile are computed, and the best performing threshold was then used as a cutoff.

### Lentivirus shRNA knockdown

The VSV-G pseudotyped lentivirus containing MAP7D3 shRNAs were purchased from the RNA Technology Platform and Gene Manipulation Core at Academia Sinica, Taiwan. A total of 10,000 cells were seeded in a 12-well culture plate and infected with the lentivirus for 48 h. Following the infection, puromycin selection was performed for 48 h to identify surviving clones. Western blotting analysis was then conducted on the harvested clones to assess MAP7D3 expression.

### Statistical analysis

The Student t-test was used to analyze the difference between two sets of data. The Log-rank test was applied to compare the Kaplan-Meier survival curves between two patient groups. To estimate the hazard ratio between the groups, the Cox proportional hazards model was employed. Additionally, the one-way ANOVA was used to compare means among more than two groups, while Pearson’s Chi-squared test was used to evaluate differences between observed and expected values in specific groups. A P-value less than 0.05 was considered statistically significant in all tests.

## Discussion

This study aimed to investigate the role of MAP7D3 in regulating TNBC progression and metastasis using the isogenic pairs of TNBC cells with low and high metastasis potential.

Our findings revealed that MAP7D3 was significantly overexpressed in highly metastatic TNBC cells, IV2 and 468-LN, compared to their respective parental cells (Fig. 1). Knockdown of MAP7D3 expression effectively suppressed in vitro metastasis traits of highly metastatic TNBC cells including cancer cell migration, invasion, and anchorage-independent growth (Fig. 2) and sensitized TNBC cells to standard chemo drug treatment such as docetaxel and gemcitabine (Fig. 3). Moreover, MAP7D3 depletion could significantly retard primary tumor growth and inhibit lung metastasis capability of metastatic TNBC cells in the orthotopic breast cancer mouse model and tail-vein injection mouse model, respectively (Fig. 4). We established the clinical relevance of MAP7D3 using multiple clinical datasets and found that high expression of MAP7D3 expression was highly associated with TNBC subtype (Fig. 5A) and poor survival outcomes of TNBC patients (Fig. 5C). Analysis of our in-house TNBC cohort samples indicated that lymph node metastatic tumors exhibited a greater expression of MAP7D3 than their corresponding primary tumors (Fig. 5B). Our research has identified the novel role of MAP7D3 in regulating the progression of triple-negative breast cancer (TNBC). This finding emphasizes the potential of targeting MAP7D3 as a therapeutic strategy for TNBC patients, thereby highlighting its value as a unique prognostic biomarker for patients with TNBC.

A previous study has shown that MAP7 and MAP7D3 function similarly as microtubule-tethered kinesin-1 activators, which regulate kinesin-1 recruitment to promote kinesin-1-dependent mitochondria distribution [18]. Additionally, MAP7D3 has been found to regulate microtubule assembly and stability [14]. Kikuchi et al. demonstrated that MAP7 and MAP7D1 could bind microtubules to facilitate microtubule remodeling, cell adhesion, and migration in HeLa cells [12]. Our research provides evidence that MAP7D3 is associated with the aggressive phenotype of highly metastatic TNBC cells, IV2 and 468-LN, which were derived MDA-MB-231 and MDA-MB-468, respectively (Fig. 1). Since patients with TNBC are usually treated with conventional chemotherapy as first-line therapy, they frequently develop resistance to the treatment [4]. Our study demonstrated that depletion of MAP7D3 expression in metastatic TNBC cells significantly increased their sensitivity to docetaxel and gemcitabine treatment (Fig. 3A-H), which are widely used as first-line chemo drugs for TNBC patients. This indicated that MAP7D3 expression levels could serve as a promising biomarker for predicting treatment response in TNBC patients who receive first-line therapy with these drugs. Previous studies have demonstrated a strong correlation between chemoresistance of breast cancer cells and the cancer-initiating cell (CICs) properties [19]. By analyzing CICs-related and chemoresistance-related protein expressions in MAP7D3-depleted metastatic TNBC cells, we found that the inhibition of MAP7D3 led to impaired expression of ABCG2, integrin α6, ALDH1A1, and Sox2, all of which are related to CICs and chemoresistance (Fig. 3I). Thus, our study provides the first evidence that inhibition of MAP7D3 could reduce the CICs marker expressions, thereby increasing the chemosensitivity of TNBC cells.

The dynamics of microtubule (MT) is critical in the signaling cascaded mediated by small GTPases which control actin polymerization and lamellipodia protrusions to drive cell migration and invasion [20]. Rac1, a small GTPase, is known to promote breast cancer lung metastasis [21] and induce chemoresistance of breast cancer [22]. Although MAP7D3 is a microtubule-binding protein, its association with Rac1 activity remains unclear. Here, we provided evidence that inhibition of MAP7D3 resulted in a reduction of Rac1 activity in metastatic TNBC cells. (Fig. 3J), which in part, providing the molecular basis to explain why MAP7D3 depletion led to suppression of metastasis-related traits and chemoresistance of metastatic TNBC cells. In addition, the mouse study confirmed that MAP7D3 expression was essential for TNBC progression that inhibition of MAP7D3 suppressed primary tumor growth and distant lung metastasis of metastatic TNBC cells (Fig. 4). Thus, we identified the novel oncogenic function of MAP7D3 in enhancing Rac1 activity and CICs properties to promote TNBC progression and metastasis.

To our knowledge, the relationship between the expression of MAP7D3 and molecular subtypes of breast cancer and survival outcomes in TNBC patients has not been explored previously. In this study, we utilized the UCSC Xena platform to analyze the TCGA breast cancer dataset and found that MAP7D3 expression was significantly correlated with TNBC patients (Fig. 5A). High expression of MAP7D3 was also strongly associated with poor survival outcomes in TNBC patients (Fig. 5C), particularly in LN-positive patients who demonstrated the worst overall survival and recurrence-free survival rates (Fig. 5C). Moreover, our investigation of paired primary tumors and LN metastatic tumors revealed that 63.8% of TNBC patients exhibited higher expression of MAP7D3, which was in line with our cell-based experiments (Fig. 1G J).

## Conclusions

Our investigation has provided new insights into the clinical significance and molecular basis of MAP7D3 in metastatic TNBC, thereby contributing to the current knowledge of MAP7D3-mediated signaling pathways associated with metastasis. Our study also highlights the potential of targeting MAP7D3 expression to combat chemoresistance in metastatic TNBC cells. In future studies, investigating the regulation of MAP7D3 and its interactions with other proteins may facilitate a better understanding of its oncogenic role in regulating TNBC metastasis. Ultimately, developing inhibitors to target MAP7D3 may offer a promising therapeutic strategy for overcoming drug resistance and preventing metastasis in TNBC.

### Electronic supplementary material

Below is the link to the electronic supplementary material.

Supplementary Material 1

## Data Availability

All data generated or analyzed in this study are included in this article and supplementary information file. All materials are available from corresponding author on reasonable request.
