# Supplementary material for: MAP7D3, a novel prognostic marker for triple-negative breast cancer, drives cell invasiveness and cancer-initiating cell properties to promote metastatic progression
Source: Biol Direct. 2023 Aug 7;18:44. doi: 10.1186/s13062-023-00400-x (PMC10405500; doi:10.1186/s13062-023-00400-x)

**Chemicals, reagents, antibodies used in this study.**

| **Chemo drug** | | | |
| --- | --- | --- | --- |
| **Name** | **Company** |  |  |
| Taxotere Docetaxel | Sanofi Taiwan Co., Ltd |  |  |
| Gemmis ® Gemcitabine HCL | TTY Biopharm Co., Ltd |  |  |
|  |  |  |  |
|  |  |  |  |
| **Name** | **Dilution** | **Catalog number** | **Company** |
| Anti-MAP7D1 | 1:2000 | #A303-780A | Fortis Life Sciences |
| Anti-MAP7D2 | 1:2000 | HPA051508 | Sigma-Aldrich |
| Anti-MAP7D3 | 1:2000 | #A302-148A | Fortis Life Sciences |
| Anti-GAPDH | 1:5000 | sc-32233 | Santa Cruz Biotechnology, Inc. |
| Anti-Rac1 | 1:5000 | #2456 | Cell signaling Technology |
| CD44 | 1:1000 | #96848 | Cell signaling Technology |
| Integrin α6 | 1:1000 | #3750 | Cell signaling Technology |
| ALDH1A1 | 1:1000 | #36671 | Cell signaling Technology |
| ABCG2 | 1:1000 | #42078 | Cell signaling Technology |
| EpCAM | 1:1000 | #93790 | Cell signaling Technology |
| Sox2 | 1:1000 | #3579 | Cell signaling Technology |
| Tissue microarray | | | |
| Name | Company |  |  |
| BR489 | US Biomax Inc. |  |  |
| BR1509 | US Biomax Inc. |  |  |
| BR1902 | US Biomax Inc. |  |  |
|  |  |  |  |
|  |  |  |  |
|  |  |  |  |
|  |  |  |  |

**Breast cancer GSE datasets**

The following GSE datasets were analyzed: GSE65194, GSE12093, GSE12276, GSE16391, GSE16446, GSE16716, GSE17705, GSE17907, GSE18728, GSE19615, GSE20194, GSE690321, GSE11121, GSE20271, GSE2034, GSE20685, GSE1456, GSE20711, GSE21653, GSE3494, GSE25066, GSE2603, GSE26971, GSE29044, GSE31448, GSE31519, GSE36771, GSE78958, GSE32046, GSE37946, GSE2790, GSE41998, GSE42568, GSE43358, GSE43365, GSE45255, GSE4611, GSE46184, GSE48390, GSE50948, GSE5327, GSE58812, GSE61304, GSE6532, GSE7390, GSE76275, and GSE9195. All above GSE datasets were analyzed using KM plotter platform.


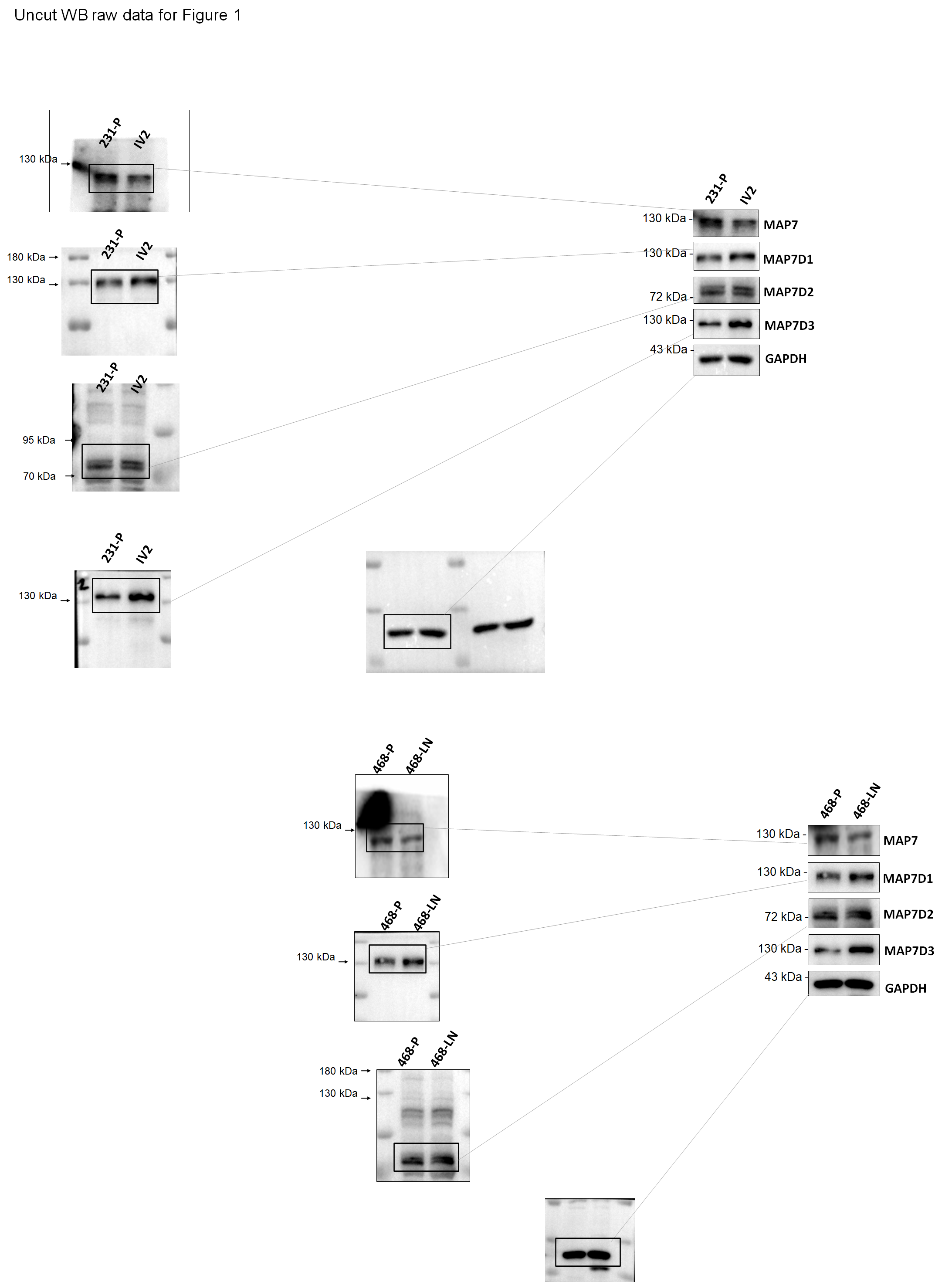


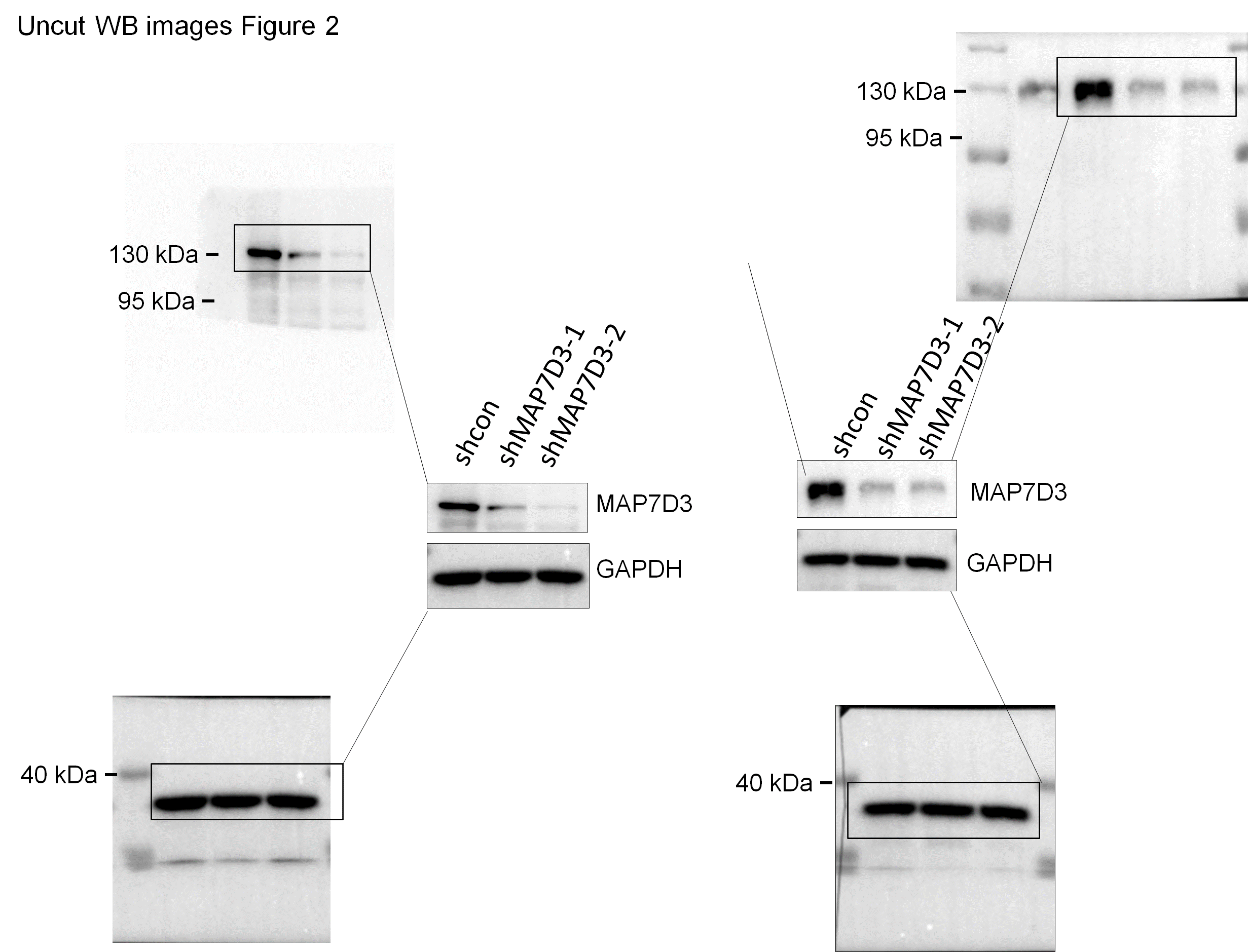


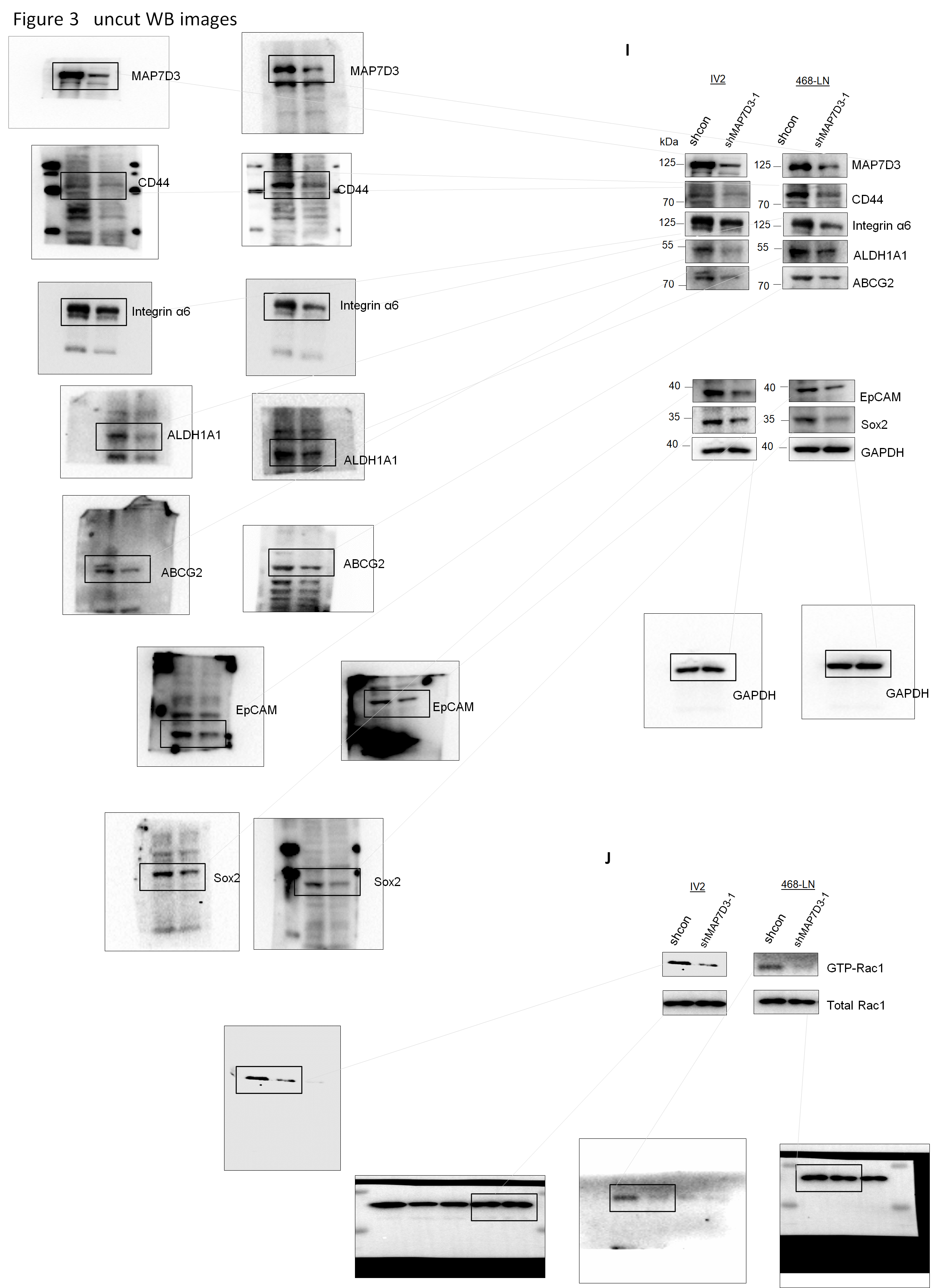

Supplement: Supplementary file 1 — Supplementary Material 1 [file 13062_2023_400_MOESM1_ESM.docx]
